# Evaluation of the Effects of Isolated Lignin on Cellulose Enzymatic Hydrolysis of Corn Stover Pretreatment by NaOH Combined with Ozone

**DOI:** 10.3390/molecules23061495

**Published:** 2018-06-20

**Authors:** Shuo Fang, Wenhui Wang, Shisheng Tong, Chunyan Zhang, Ping Liu

**Affiliations:** 1 College of Food Science and Nutritional Engineering, China Agricultural University, Beijing 100083, China; shuofangssdd@163.com (S.F.); wwhui123321@163.com (W.W.); 18333189687@163.com (C.Z.); 2 Bio-Pharmaceutical College, Beijing City University, Beijing 100094, China; shishengt@163.com

**Keywords:** alkaline combined with ozone, pretreatment, enzymatic hydrolysis, lignin

## Abstract

In this experiment, corn stover was treated with optimal combined pretreatment conditions: 2% NaOH at 80 °C treated 2 h combined with initial pH 9 at the ozone concentration of 78 mg/mL treated 25 min. The effect of lignin removal rate on the enzymatic hydrolysis degree of cellulose during the treatment process was studied. At the same time, the lignin in the optimal pretreated corn stover was separated and extracted by enzymatic acidolysis, and its structure and connection were characterized. The results showed that the alkali combined with ozone pretreatment improved the enzymatic hydrolysis degree of the cellulose while exfoliating and degrading the macromolecular lignin into small molecules. The stable crosslink structure of the lignin-cellulose-hemicellulose was destroyed, and the lignocellulosic structure changed in favor of the enzymatic hydrolysis of the cellulose.

## 1. Introduction

Growing energy demands and consumption of fossil fuels stimulate the search for biomass lignocellulose production fuels and chemicals [1,2]. Biomass lignocellulose can produce a variety of products of the biorefinery process [3]. However, due to the complex structure of lignocellulose, where hemicellulose and lignin form a dense network-like structure that envelops cellulose, impeding its contact with enzymes, and the fact that the highly crystalline structure of cellulose itself makes it difficult to use directly [4,5]. Therefore, pretreatment of raw materials to increase the utilization efficiency of various components is the basis of the biotransformation of lignocellulose. A large number of experiments in the early stage of the lab have found that alkali combined with ozone pretreatment of corn stover is beneficial to the enzymolysis of cellulase, and the alkali combined with ozone pretreatment conditions that are the most conducive to promoting the enzymatic hydrolysis of corn stover are obtained, which is treatment with 2% NaOH at 80 °C for 2 h and initial pH 9 ozone treatment for 25 min. However, the mechanism of the effect of lignin structures change on the enzymatic hydrolysis of cellulose after pretreatment is not clear.

Alkaline pretreatment is an effective method of removing lignin from lignocellulosic materials and increasing the accessibility of enzymes to lignocellulose [6]. Alkaline pretreatment methods are generally performed at ambient temperature and pressure. The most common alkali reagents are the hydroxy derivatives of the sodium, potassium, calcium, and ammonium salts. Among these hydroxy derivatives, sodium hydroxide is considered to be the most effective. Alkaline pretreatment causes lignin structure to be destroyed, cellulose swells, cellulose crystallinity decreases, and hemicellulose solvates [7]. Menezes et al. optimized the alkaline pretreatment coffee grounds and found that using 4% (*w*/*v*) sodium hydroxide solution to 25 min are best [8]. Taher et al. pretreatment of potato peel residues with a 1% (*w*/*v*) sodium hydroxide solution at 121 °C for 30 min reduced the lignin content from 4.7% to 1.1%, and the saccharification yield increased from 20% to 58% [9].

Ozone is the strongest oxidant in nature, and its oxidation-reduction potential is second only to fluorine. The ozone oxidation ability is strong and can react with many organic substances or functional groups. However, there is still no affirmative research conclusion of the mechanism of ozone oxidation reaction. It is generally considered that there are two ways for ozone to react with organic matters: one is that ozone directly reacts with organic substances in the water in the form of oxygen molecules; the other is that in the alkaline condition, ozone decomposes into water to produce highly oxidized hydroxyl radicals, and indirect oxidation reactions occur [10]. Ozone can strongly react with substances containing conjugated double bonds and high electron density functional groups. Lignin in stover contains a large number of carbon-carbons double bonds, aromatic rings, and other electron clouds dense groups and thus is easy to degrade by ozone. Travaini et al. found that bagasse after ozone pretreatment produces xylitol, lactic acid, formic acid, acetic acid, and other degradation products [11]. According to Panneerselvam et al., degradants produced by ozone pretreatment significantly inhibit enzymolysis [12,13]. Sannigrahi et al. processed three biomass feedstocks (Loblolly pine, Sweetgum, and Miscanthus) with two-step ozone pretreatment. The results showed that ozone treatment alone can generate oxidative biomass fragments, while two-step ozone pretreatment (5% ozone at 30 °C treated 60 min combined with ethanol/water solution treatment under different conditions) led to significant delignification and preserved most of the carbohydrates in the three biomass feedstocks [14]. Lee et al. studied both pretreatment techniques of ozone and autohydrolysis and found that the autohydrolysis treatment improved the reactivity of cellulose, hemicellulose, and lignin to ozone, which further indicated that ozone treatment works better combined with other pretreatment methods [15].

Research has shown that lignin may affect the enzymatic hydrolysis of cellulose in two ways: (1) Lignin acts as a physical barrier, limiting the accessibility of cellulose and enzymes, and (2) Lignin can interact irreversibly with cellulase through hydrophobic interactions [16]. Recent studies have found that lignin content, lignin functional group composition, and physical distribution affect the efficiency of enzymatic hydrolysis [17]. Yuan et al. reported that the efficiency of enzymatic hydrolysis increases with the decrease of lignin content after ionic liquid pretreatment [18]. Some scholars have found that lignin isolated from hardwood pretreated at higher severities resulted in serious inhibition in the enzymolysis of Avicel [19]. In short, the effect of lignin on the enzymolysis of cellulase remains unclear due to non-specific binding of lignin or physical barriers or natural chemical differences, but the study of the mechanism of the interaction between cellulose enzymolysis and lignin is the basis for optimizing the pretreatment conditions [20]. 

At present, in the research on the effects of pretreatment methods on the content and structure of lignin in lignocellulose, most of the physical and chemical structure characterizations are devoted to the determination of the overall structure of the remaining part after pretreatment of lignocellulose, but the isolated lignin sample can represent the true structure of the original lignin in lignocellulose and pretreated lignin. Under the optimum alkali combined with ozone pretreatment condition, the lignin structure in the stover changes to some extent so that the enzymolysis effect of cellulose is optimized. In order to observe the effects of alkali treatment and ozone treatment on the lignin structure and the linkage of lignin with other carbon-water mixtures and further clarify the mechanism of lignin removal to promote the enzymatic hydrolysis of cellulose, in this study, corn stalks were used as raw materials, and the lignin content and cellulose enzymolysis degree in corn stover without pretreatment, optimal alkali combined with ozone pretreatment, optimal single alkali treatment, and optimal single ozone treatment were determined and used. The lignin in the remaining stover after pretreatment was separated and purified by enzymatic hydrolysis, and the lignin in the solution was separated by a two-step precipitation method. The lignin preparations isolated from the remaining stover after pretreatment was characterized by GPC, FTIR, ^1^H-NMR, and TAG-DTA [2,21,22,23].

## 2. Materials and Methods 

### 2.1. Raw Material

The raw materials used in the experiment were corn stalks from a farm in Siping, Northeast China. The raw materials were crushed by a pulverizer, sieved through a 60-mesh sieve, extracted with toluene:ethanol = 2:1 for 3 h, and dried to a constant weight for later use. The content of cellulose, hemicellulose and lignin in corn stover was determined by two-step acid hydrolysis method to be 40.83%, 21.97%, and 28.15%, respectively [24].

### 2.2. Separation of Remaining Lignin after Pretreatment

We took the same quality (20 g) pulverized, dewaxed, and dried corn stalk raw materials separately for ozone pretreatment alone (initial pH 9 ozone treatment for 25 min), alkaline pretreatment alone (2% NaOH treatment at 80 °C for 2 h), and alkali combined with ozone pretreatment (treatment with 2% NaOH at 80 °C for 2 h and initial pH 9 ozone treatment for 25 min). Three kinds of different pretreated and non-pretreated corn stalks were put into four stainless steel ball mill tanks, respectively, and the pellets with the diameter of 1 cm were added to each ball mill jar with the ratio of pellets of 1:20. After sealing, they were fixed in a planetary high-energy ball mill. The mode was as follows: milling time: 30 min, intermittent time: 10 min, rotation speed: 340 rpm/min, cumulative ball grinding time: 2 h [25].

We weighed the sample with the mass M_1_ (g) ball milled into a 250 mL Erlenmeyer flask and added an acetic acid-sodium acetate buffer solution with a pH of 4.8 at a solid-liquid ratio of 1:20. We then added 10% volume of enzyme solution, containing 3000 units of carboxymethyl cellulase activity per mL of enzyme solution. The conical flask was placed in a 40 °C water bath shaker for 48 h at 200 rpm. The enzymolyzed mixture was passed through a 3000-mesh filter cloth, and the upper residue was washed with hydrochloric acid having a pH of 2. After the residue was frozen at −4 °C overnight, the crude lignin was digested by freeze-drying for 48 h and weighed and designated as M_2_ (g). Freeze-dried crude lignin was weighed in a single-necked round-bottomed flask, and an acidic dioxane aqueous solution was added at a solid-to-liquid ratio of 1:25 (dioxane: 0.01 mol/L hydrochloric acid = 85 mL:15 mL, *v*/*v*). The mixture was placed in a constant temperature water bath at 87 °C and refluxed for 2 h. After cooling to room temperature, the mixture was centrifuged at 4000 rpm for 10 min to collect the supernatant. The residue was washed with a neutral dioxane-water solution until the washings were clear, freeze-dried for 48 h, and weighed and designated as M_3_ (g). The washing solution was combined with the supernatant, neutralized with NaHCO_3_ solution dropwise, and evaporated to 30 mL under reduced pressure at 35 °C. The concentrated solution was added dropwise to 1 L of acidic (6 mol/L HCl acidified pH = 2.0) deionized water to precipitate the enzymatically digested lignin (EMal), which was lyophilized and weighed as L [26].

### 2.3. Enzymatic Hydrolysis

Then, 0.2 g of pretreated stover samples were placed in 100 mL Erlenmeyer flask, and 10 mL of acetate buffer (0.1 mol/L, pH 4.8) were added, which was prepared by sterile water and contained 40 μg/mL tetracycline, 30 μg/mL cycloheximide, and 40 μL xylanase solution. The mixture was incubated in a shaking bath (120 rpm) at 70 °C for 24 h. After the reaction, it was cooled down to room temperature, we added 40 μL cellulase 30 μL β-glucosidase, and then it was incubated at 50 °C for 72 h. Cycloheximide can inhibit the DNA translation of eukaryotes to stop the cell growth or even die. The purpose of adding cycloheximide and tetracycline hydrochloride was to inhibit the growth of microorganism that influenced the pH value during the enzymatic process and affected enzyme activity. Enzymatic hydrolysate was filtered through 0.22 μm membrane and then analyzed by HPLC (Agilent, Palo Alto, CA, USA) (company, city, state abbre. if USA or Canada, country) to determine the glucose content and calculate the cellulose enzymolysis degree.

### 2.4. Determination of Lignin Content

Two-step acid hydrolysis. The first step: Accurately weighed 0.300 g of the test sample into a test tube, added 3.0 mL of 72% sulfuric acid solution, and mixed by vortexing. It was placed the tube in a 30 °C water bath for 60 min and mixed once every 5–10 min. The second step: We diluted the sulfuric acid concentration to 4% after the water bath was completed and put it in a retort at 121 °C for 1 h. After filtration, the absorbance of the filtrate was measured at 320 nm with 4% sulfuric acid as the control by UV-Vis spectrophotometry to calculate the content of acid soluble lignin (ASL). The filter residue was dried to constant weight and used to determine the content of acid insoluble lignin (AIL). The acid-insoluble lignin determined by this method contains acid-insoluble ash and acid-insoluble protein, which were negligible due to their small content. We repeated the measurement three times for each sample and used the average as the result of the calculation.

$$\mathrm{AIL}\;(\%)=OD_{320}\times V\times n\times 100/(\varepsilon \times 300)$$

$$\mathrm{ASL}\;\left(\%\right)=\left(m_{1}-m_{2}\right)\times 100/0.3$$

where *V* was the total reaction system volume (mL), *m* was the test sample dry matter content (mg), *n* was the dilution times, *ε* was the absorbance coefficient of corn stover at wavelength of 320 nm (30 L/g·cm), *m*_1_ was the quality of the core funnel (mg), and *m*_2_ was the total mass of the sand funnel and filter residue (mg).

### 2.5. Determination of Glucose by HPLC

Glucose content was measured by the Agilent 1200.The HPLC configuration was: column: Rezex ROA and corresponding guard column; detector: differential detector; sample injection volume: 20 μL; mobile phase: 0.005 M H2SO4, 0.22 μm filter membrane, degassing; flow rate: 0.6 mL/min; column temperature: 65 °C. Glucose retention time: 9.812 min.

### 2.6. Structural Characterization of Isolated Lignin

#### 2.6.1. Gel Permeation Chromatography (GPC) Analysis

The average molecular weight (Mw), number average (Mn), and polydispersity (Mw/Mn) of the lignin samples were determined by GPC (Agilent, Palo Alto, CA, USA). The lignin samples were dissolved in 1 mL tetrahydrofuran solution. Sample solutions were syringe filtered through 0.22 μm nylon filters prior to analysis. The GPC configuration was as follows: column, Plgel Mixed-D (300 × 7.5 mm, Agilent, Palo Alto, CA, USA); eluent: chromatographically pure THF; flow rate: 1 mL/min; injection volume: 20 μL. The gel column, which measured the average molecular mass of the lignin sample, was always at a constant outside temperature.

#### 2.6.2. Fourier Transform Infrared Spectroscopy (FTIR) Analysis

The samples were placed in an oven at 50 °C for 24 h to remove moisture. Then, a 10 mg dry sample was mixed with 200 mg KBr, manually ground in an agate mortar, and pressed at 20 MPa for 2 min in oil pressure. The tablets were placed on a sample rack for FTIR spectra spectroscopy (Agilent, Palo Alto, CA, USA), and the spectra were recorded between 4000 and 400 cm^−1^. The PerkinElmer Spectrum (PERKINELMER, Waltham, MA, USA) and Origin software (OriginLab, Northampton, MA, USA) were used for data analysis.

#### 2.6.3. ^1^H Nuclear Magnetic Resonance (NMR) Analysis

The one-dimensional liquid nuclear magnetic spectrum ^1^H of the lignin preparation was measured by the Bruker AVIII 700 MHz spectrometer (Agilent, Palo Alto, CA, USA) in the Fourier transform mode at 100.6 MHz. A 25 mg sample was dissolved in 1 mL DMSO-*d*_6_. Spectra were acquired with 30° pulses using an acquisition time of 3.98 s and a relaxation delay of 1 s 25 °C scans 128 times, and the pulse width was 13.6 μs. 

#### 2.6.4. Thermo Gravimetric (TG) and Differential Thermal Gravity Analysis (DTA)

Thermo gravimetric analysis (TGA) was carried out on a shimadzu DTA-60 (Beijing Henven Scientific Instument Factory, Beijing, China). The samples (5–10 mg) were heated at a heating rate of 10 °C/min from 40 to 600 °C with a N_2_ flow rate of 30 mL/min. Before the measurement, the sample was baked in an oven at 105 °C for 2 h to constant weight. The solid A1203 was used as a reference.

## 3. Results and Discussion 

### 3.1. The Effect of Lignin Removal Rate Changes after Alkali-Ozone Combined Pretreatment of Corn Stover on the Cellulose Enzymatic Hydrolysis 

**Figure 1:**
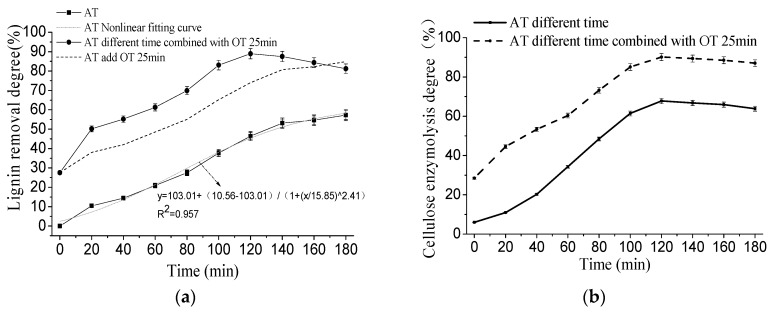
Effect of NaOH treatment on the lignin content of corn stover in alkali combined with ozone pretreatment. (**a**) Effect of NaOH treatment on the lignin removal degree of corn stover in alkali combined with ozone pretreatment; (**b**) effect of NaOH treatment on the cellulose enzymolysis degree of corn stover in alkali combined with ozone pretreatment.

The effect of NaOH treatment on the removal rate of lignin of corn stover is shown in Figure 1. The removal rate of lignin increased with the treatment time with alkaline treatment (AT) alone, and its nonlinear fitting showed that R^2^ = 0.957, indicating that the fitting equation can well predict the change of lignin content with time in alkali treatment alone. The cellulose enzymolysis degree increased first and then decreased with the time of alkali treatment alone, indicating that the removal of lignin in the stover during this treatment stage was not the only factor limiting the enzymatic hydrolysis of cellulose. With the extension of processing time, the removal rate of lignin in stover treated with alkali treatment at different times combined with ozone treatment (OT) increased first and then decreased. The maximum lignin removal rate was 88.92% when treated with alkali for 120 min combined with ozone treatment for 25 min. When the treatment time was further extended, the lignin removal rate decreased, which was consistent with the changing trend of cellulose digestion. The reason may be that the long-term treatment of NaOH at high temperature will lead to the exposure of free hydrogen bonds in cellulose, thereby increasing the affinity with water and accumulating stover particles agglomerates. Therefore, the reaction area between stover and ozone was reduced, which affected the lignin removal effect during the ozone pretreatment and subsequent enzymolysis effect.

**Figure 2:**
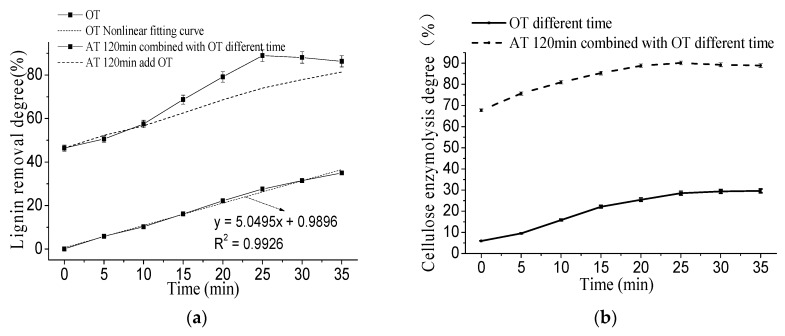
Effect of ozone treatment on the lignin content of corn stover in alkali combined with ozone pretreatment. (**a**) Effect of ozone treatment on the lignin removal degree of corn stover in alkali combined with ozone pretreatment; (**b**) effect of ozone treatment on the cellulose enzymolysis degree of corn stover in alkali combined with ozone pretreatment.

The effect of ozone treatment on the removal rate of lignin of corn stover is shown in Figure 2. It can be seen from the figure that there was a strong linear relationship between the removal rate of lignin and the treatment time when treated with ozone alone (R^2^ = 0.993), but the removal efficiency was not as good as the alkali treatment. Ozone treatment can increase the enzymolysis efficiency of cellulose, and the increase range is consistent with the trend of lignin removal rate. The reason may be that the reduction of lignin significantly improved the ability of stover particles to adsorb cellulase. After alkali treatment for 120 min combined with ozone treatment, the lignin removal rate increased first and then decreased with the prolonged treatment time. The reason for this phenomenon was that, in the process of alkaline ozone treatment, peeling-adsorption-stripping occurred during lignin removal. That was, at a certain pretreatment intensity, the removal rate of lignin was high. However, as the strength of pretreatment was further enhanced, the removal of lignin caused some impurities to adsorb on the surface of the corn stover, resulting in a decrease in the removal rate of lignin. When the pretreatment intensity continued to increase to a certain extent, the impurities adsorbed around the lignin were removed, thereby increasing the lignin removal rate. During the ozone treatment, the change trend of cellulose enzymolysis degree was consistent with the removal rate of lignin. After alkali treatment for 120 min combined with ozone treatment for 25 min, with the prolonged treatment time, the lignin removal rate and the enzymolysis degree of cellulose decreased. The reason may be that the proportion of lignin is difficult to degrade in the remaining stover is increased, which limits the contact of the cellulase with the internal cellulose and thus inhibits the enzymatic hydrolysis.

### 3.2. Extraction Rate of Lignin in Corn Stover after Alkali Combined with Ozone Pretreatment

The extraction rate of the isolated lignin preparations is shown in Table 1. As can be seen from the table, the extraction rate of the remaining lignin in the stover after pretreatment was higher than that of untreated. The extraction rate of alkali combined with ozone pretreatment was the highest, which was 84.83%, indicating that combined pretreatment can effectively open the complex and compact network structure of lignocellulose. Coarse lignin is likely to entrain other components, making the structure characterizations unable to accurately reflect the effect of structural changes in lignin on enzymatic hydrolysis of cellulose. Therefore, it is necessary to purify the crude lignin. 

The extraction rate of purified lignin preparation of untreated corn stover was 4.64% of the original lignin. The extraction rate of pure lignin after pretreatment of straw was higher than that of untreated, and the extraction rate of alkali combined with ozone pretreatment yielded the highest extraction rate of 8.56%. It was proven that the pretreatment had a destructive effect on the connection between lignin and other components. The destruction caused the exposed cellulose enzymolysis sites were fully bound to the enzyme molecules, increasing the cellulose enzymolysis degree. 

### 3.3. Effect of Lignin Molecular Weight Changes on Cellulose Enzymatic Hydrolysis after Alkali Combined with Ozone Pretreatment

**Figure 3:**
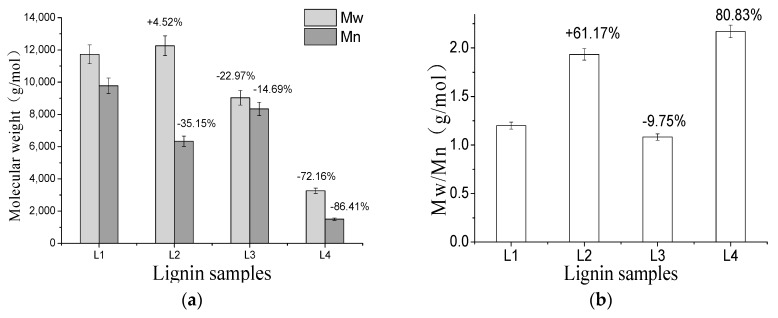
Comparative analysis of molecular weight of lignin in different pretreatment corn stover. Note: L_1_ (untreated stover lignin), L_2_ (alkaline treated stover lignin), L_3_ (ozone treated stover lignin), L_4_ (alkaline combined with ozone treated stover lignin). (**a**) Molecular weight of lignin samples; (**b**) Mw/Mn of lignin samples.

The results of the number average (Mn), average molecular weight (Mw) and polydispersity (Mw/Mn) obtained for lignin samples extracted from the remaining stover after pretreatment are shown in Table 2. Pretreatment and untreated molecular weight ratios are shown in Figure 3.

The results showed that the molecular weight distribution of lignin in the remaining stover after pretreatment was in the range of 3264–12255 g/mol, but the molecular weight of the untreated lignin (11,725 g/mol) was higher. Because the lignin structure in the untreated stover was closer to the lignin structure in the raw material. In the alkaline treatment alone, the alkali solution caused the lignin macromolecules to peel off through the saponification. At the same time, a part of the lignin fragments aggregated together and form a macromolecule through a polymerization reaction at a relatively high temperature in the alkaline environment. Therefore, the average molecular weight of the lignin preparation was increased by 4.52%. The main role of ozone treatment was to degrade lignin macromolecules as small molecules so that the average molecular weight of lignin decreased by 72.16% after alkali combined with ozone pretreatment. In addition, it was found that the polydispersed coefficient of lignin isolated from untreated stover was 1.200. Compared with the results, the polydispersed coefficient of lignin isolated after ozone treatment alone increased by 61.17%, the alkali treatment alone decreased by 9.75%, and the alkali combined with ozone treatment increased by 80.83%. This was due to the degradation of lignin in plant cell walls to small-molecule organic acids after ozone treatment, and showed that the lignin structure obtained by ozone pretreatment was not homogeneous [27].

### 3.4. FTIR Analysis of Lignin after Alkali Combined with Ozone Pretreatment and Its Effect on Cellulose Enzymolysis 

**Figure 4:**
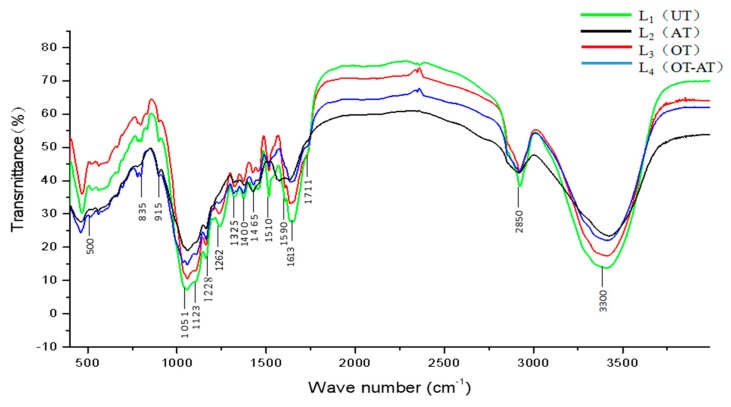
The Fourier Transform Infrared Spectroscopy (FTIR) spectra of lignin obtained in the residue of different pretreatment of corn stover. Note: L_1_ (untreated stover lignin), L_2_ (alkaline treated stover lignin), L_3_ (ozone treated stover lignin), L_4_ (alkaline combined with ozone treated stover lignin).

The effect of different pretreatments on the structure destruction of corn stover and the degree of lignin removal was different. In order to analyze the effects of different pretreatment methods on the monomer composition, bond structure of lignin, and the effect of lignin on the bonding of cellulose and hemicellulose, the FTIR of the lignin separated from untreated stover and the three remaining stover after pretreatment were measured [28]. The results are shown in Figure 4.

By comparing the FTIR of the lignin in the remaining stover in the untreated (L_1_), alkaline treatment (L_2_), ozone treatment alone (L_3_), and alkaline combined with ozone pretreatment (L_4_), it was found that the four spectra were similar. This showed that the main chemical functional groups of lignin in the three pretreated corn stalks were the same as those in the untreated corn stover. Comparing the characteristic absorption of four lignin preparations at wavenumbers 1613, 1510, 1465, and 1400 cm^−1^ resulting from the vibration of the aromatic lignin skeleton, it was found that the four lignin samples have similar characteristic absorptions. This shows that the four lignin samples have similar aromatic ring skeletons. The untreated absorbance at these four wave numbers were lower than the three pretreatments, indicating that the lignin underwent structural changes during the pretreatment process, resulting in monomers with strong infrared absorption. 

1590 cm^−1^ is the vibrational absorption of C-C stretching on the lignin aromatic ring. L_2_ did not have a characteristic absorption peak here, indicating that the saponification occurs at the α-position of the methylene group in the side chain of the lignin during the alkali treatment, and the ether bond broke to generate the methylene oxime intermediate, resulting in acyclic α-*O*-4 structural unit separation. At the same time, the phenolic hydroxyl group (β-*O*-4) dehydrogenated H^+^ to form phenoxide anion, and the cleavage of non-phenolic β-*O*-4 played an important role in the dissolution of lignin during the subsequent ozone treatment. 1711 cm^−1^ is the characteristic absorption of non-conjugated carbonyl in lignin degradation products. It can be found that there was no absorption peak at this point in the alkali treatment because alkali treatment dissolved and removed the lignin from corn stover, thereby reducing the concentration of lignin in the stover. Ozone treatment increased the absorbance at this wave number compared to untreated ones, indicating that ozone treatment alone degraded lignin to produce more non-conjugated carbonyl compounds. The result was consistent with the FTIR result of lignin obtained from alkali treatment of sugarcane bagasse by Naron et al. [21]. In the FTIR of the lignin obtained for the alkali combined with ozone treatment, the non-conjugated carbonyl at the wave number of 1711 cm^−1^ produced a strong vibrational absorption of the C=O double bond indicating that the lignin in the remaining stover after alkali treatment was more easily degraded during the ozone treatment. Absorption at 1325 cm^−1^ is caused by C-O respiration on the aromatic ring of lilac, and absorption at 1123 cm^−1^ is generated by internal deformation of the aromatic ring of lilac. The absorption of L_2_ in these two wave numbers was the strongest, indicating that the effect of alkali treatment on syringyl was less, and ozone had a greater impact on it. Absorption at 1262 cm^−1^ results from the C=O respiratory stretching vibrations on the guaiac wood ring. The absorption of L_2_ at 1262 cm^−1^ was the strongest, which was consistent with the discovery of Toledanoet et al. [29]: high-molecular-weight lignin samples contained more guaiacyl units, and the 5-5′-linkages formed between the guaiac units did not break during the alkaline treatment alone [30]. From this, it can be seen that there were differences in the proportions of lilac units and guaiacyl units in the four lignin samples. The absorption band at 1051 cm^−1^ is generated by the bending vibration of hydroxyl groups in lignin. L_2_ had the largest absorbance at this wave number, followed by L_4_, and L_3_ was lower. It showed that the alkali treatment produced a condensation reaction with the alcoholic hydroxyl group of lignin to produce aldehyde-based compounds, while the new ecological oxygen atoms produced by ozone decomposition reacted only with the hydroxyl groups on the surface of lignin.

### 3.5. ^1^H NMR Analysis of Lignin after Alkali Combined with Ozone Pretreatment and Its Effect on Cellulose Enzymolysis 

**Figure 5:**
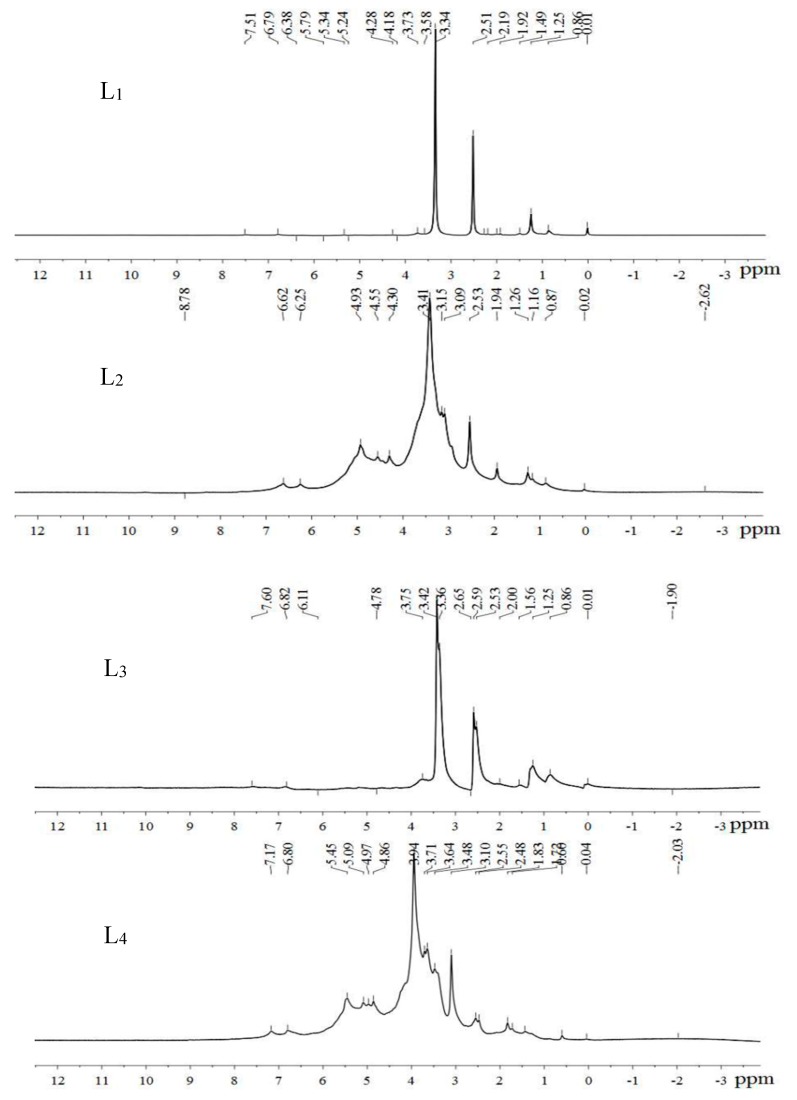
^1^H-NMR spectra of lignin obtained in the residue after different pretreatment of corn stover. Note: L_1_ (untreated stover lignin), L_2_ (alkaline treated stover lignin), L_3_ (ozone treated stover lignin), L_4_ (alkaline combined with ozone treated stover lignin).

Figure 5 shows the ^1^H NMR spectra of lignin remaining in the stover after untreated (L_1_), alkaline treatment alone (L_2_), ozone treatment alone (L_3_), and alkali combined with ozone pretreatment (L_4_). As can be seen from the figure, the four lignin preparations were guaiacyl-syringol type lignin (GS type lignin).

Observing Figure 5, we found that L_2_ chemical signal appeared at a chemical shift of δ 9.45 ppm. Herewas a chemical signal generated by -CH=CH-CHO on the benzene ring, indicating the methylene hydroxyl group on the benzene ring of lignin in the stover had been oxidized to generate aldehyde groups after alkali treatment alone [31]. After the ozone treatment alone, the chemical signal here disappeared, indicating that the aldehyde group was oxidized to a carboxyl group as a hydrophilic group. L_1_ had stronger chemical signals for β-*O*-4 hydrogen atoms at δ 4.28 and 4.18 ppm. L_3_ had a weak β-*O*-4 ether linkage chemical signal at δ 4.78 ppm. L_2_ showed a weak chemical signal of β-*O-*4 hydrogen at δ 4.30 ppm, and strong β-*O-*4 ether bond signal at δ 4.93 and δ 4.55 ppm. The chemical signal of the β-*O*-4 hydrogen atom of L_4_ vanished, and there was a strong chemical signal at δ 4.97 and δ 4.86 ppm. The chemical signal of the β-*O*-4 hydrogen atom of L_4_ disappeared and there was a strong chemical signal at δ 4.97 and δ 4.86 ppm. It showed that after alkali combined with ozone pretreatment, the etherification of the β-*O*-4 linkage inside lignin was destroyed, and the benzyl ether bond was etherified at the α-position of the side chain of the lignin macromolecule, which was consistent with the results of the FTIR. The two strong signals of L_1_ at δ 3.73 and 3.34 ppm are the chemical signals generated by protons in the methoxy group. After the alkali treatment, the peaks were split, and after the ozone treatment, the peaks were coupled. After the alkali combined with ozone pretreatment, the peak appeared split, and the split peaks were multiple peaks. A strong single-peak signal appeared in this region before lignin was oxidized. After being oxidized, the proton signal in this region decreased and split or coupled, indicating that the aromatic cyclomethoxy group was reduced after oxidation. L_1_, L_2_, and L_3_ had a chemical signal at the chemical shift δ 1.25 ppm, indicating that lignin contained a small amount of hydrocarbons, and L_4_ had no chemical signal here, indicating the dehydrogenation of hydrocarbons in the lignin after pretreatment. 

### 3.6. TGA-DTA of Lignin after Alkali Combined with Ozone Pretreatment and Its Effect on Cellulose Enzymolysis 

**Figure 6:**
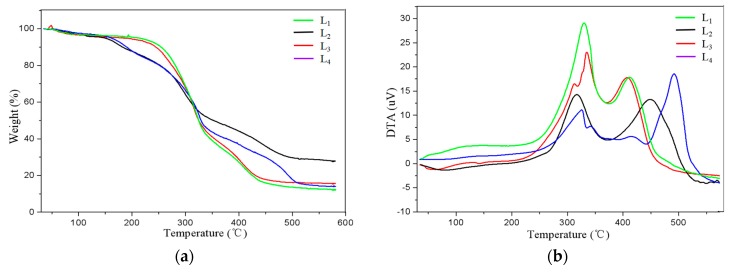
Thermo gravimetric analysis (TGA) (**a**) and differential thermal gravity analysis (DTA) (**b**) curves of lignin obtained in the residue of different pretreatment of corn stover. Note: L_1_ (untreated stover lignin), L_2_ (alkaline treated stover lignin), L_3_ (ozone treated stover lignin), L_4_ (alkaline combined with ozone treated stover lignin).

The weight loss of lignin in the pyrolysis process was mainly due to the weight loss caused by the precipitation of gases generated by a series of chemical reactions that occurred with increasing temperature. Figure 6 shows the TGA and DTA of enzymatically digested lignin in the remaining stover after untreated (L_1_), alkaline treatment alone (L_2_), ozone treatment alone (L_3_) and alkali combined with ozone treatment (L_4_). From the TGA, it can be seen that the quality of both L_3_ and L_1_ were balanced after a slight loss first, and then the loss of quality gradually decreased and tended to balance after a large loss, while the quality of L_2_ and L_4_ began to show a greater loss trend and tended to balance. Lignin was composed of three phenylpropane monomers linked by ether bonds and was rich in hydroxyl and methoxy groups. Therefore, the slight weight-loss zone that occurred during the period from 40 °C to 200 °C was mainly due to the thermal weight loss caused by the breakage of the aliphatic hydroxyl groups on the branches in the lignin structure and the formation of water and other small molecular volatile substances. Since then, due to the temperature rising lignin cracked, the sample quality dropped sharply in the range of 200 °C–330 °C, making the sample quality loss reached 37–50%. This was because the ether bond in the dominant structure of lignin broke down and the products were mainly various phenolic substances. The pyrolysis temperature of the lignin in the untreated stover was 196.4 °C, and the pyrolysis occurred at 202.3 °C in the ozone treatment alone. However, the alkali-treated and alkali combined with ozone samples began to crack at 158.8 °C, and the pyrolysis temperature was about 38 °C earlier than that of the untreated ones, indicating that the lignin extracted from the remaining stover after the combined pretreatment had loose structure and poor thermal stability. The obvious weight loss can still be seen in 330 °C–425 °C. The main reactions were the depolymerization of lignin macromolecules, the polymerization of unsaturated branched aliphatic hydrocarbons, alcohols, aldehydes, and the ring-opening reaction of benzene rings. The cleavage products at this time were mainly some long-chain esters and polycyclic aromatic hydrocarbon structures. 

425 °C–500 °C was the carbon residue burning zone, after which the quality of lignin remained basically unchanged. When the lignin reached 600 °C, the combustion ended, and the residue of the pyrolysis coked. The residual carbon values of L_1_, L_2_, L_3_, and L_4_ were 11.94%, 27.69%, 15.47%, and 13.95%, respectively. The residual carbon values of L_2_ and L_4_ increased, indicating the degradation of lignin in corn stover during the alkaline combined with ozone pretreatment. This result was in line with the Singh et al. report, i.e., under high-temperature conditions, lignin will also undergo polycondensation at the same time as degradation, resulting in a highly stable cross-linked structure and a higher residual rate [32].

From the DTA, the lignin in the untreated stover reached the maximum weight loss rate of 7.24 mg/min at 330.1 °C and 411.3 °C. Ozone pretreatment alone achieved the maximum weight loss rate of 5.83 mg/min at 334.9 °C and 407.3 °C. Alkaline pretreatment alone achieved the maximum weight loss rate of 2.96 mg/min at 317.1 °C and 447.9 °C. The alkali combined with ozone pretreatment achieves the maximum weight loss rate of 2.75 mg/min at 325.8 °C and 491.4 °C. After three pretreatments, the maximum weight loss rates of lignin in the remaining stover were lower than that of the untreated corn stalk, indicating that alkali pretreatment alone, ozone pretreatment alone, and alkali combined with ozone pretreatment all can destroy the lignin in the stover.

## 4. Conclusions

This study used the enzymatic hydrolysis method to separate lignin from pretreated corn stover. It was found that the alkali combined with ozone pretreatment made the stover swell, and the macromolecular lignin degraded into small molecules to reduce the molecular weight. The smaller the molecular weight of lignin, the lower the thermal stability of lignin. FTIR and NMR analysis showed that the β-*O*-4 ether bond between lignin units was broken, the β-β and β-5 carbon-carbon bonds were condensed, the proportion of guaiacyl and syringyl units and the contents of hydroxyl and methoxyl were decreased, which destroyed the stable cross-linking between lignin and ferulic acid. The above structural changes and breakage of linkages caused the degradation of lignin macromolecules, which changed the direction of the structure change of lignocellulose in favor of the cellulose enzymatic hydrolysis.

## Figures and Tables

**Table 1:** Extraction rate of lignin separated from different pretreated corn stover residues.

Lignin Preparations	L_1_	L_2_	L_3_	L_4_
Crude lignin extraction degree/%	59.76	78.68	63.50	84.83
Purified lignin extraction degree/%	4.64	6.66	5.64	8.56

Note: L_1_ (untreated stover lignin), L_2_ (alkaline treated stover lignin), L_3_ (ozone treated stover lignin); L_4_ (alkaline combined with ozone treated stover lignin).

**Table 2:** Weight-average (Mw) and number-average (Mn) molecular weights and polydispersity (Mw/Mn) of lignin fractions obtained by enzymatic hydrolysis of different pretreatment corn stover.

Average Molecular Weight (g/mol)	Lignin Component			
	L_1_	L_2_	L_3_	L_4_
Mw	11,725	12,255	9032	3264
Mn	9773	6338	8337	1504
Mw/Mn	1.200	1.934	1.083	2.170

Note: L_1_ (untreated stover lignin), L_2_ (alkaline treated stover lignin), L_3_ (ozone treated stover lignin), L_4_ (alkaline combined with ozone treated stover lignin).
